# Review of the speculative role of co-infections in *Streptococcus suis*-associated diseases in pigs

**DOI:** 10.1186/s13567-021-00918-w

**Published:** 2021-03-20

**Authors:** Milan R. Obradovic, Mariela Segura, Joaquim Segalés, Marcelo Gottschalk

**Affiliations:** 1 Groupe de Recherche Sur Les Maladies Infectieuses en Production Animale (GREMIP), Centre de Recherche en Infectiologie Porcine et Aviaire (CRIPA), Faculty of Veterinary Medicine, University of Montreal, 3200 Sicotte, Saint-Hyacinthe, QC J2S 2M2 Canada; 2 UAB, CReSA (IRTA-UAB), Campus de la UAB, 08193 Bellaterra (Cerdanyola del Vallès), Spain; 3 Departament de Sanitat I Anatomia Animals, Facultat de Veterinària, UAB, 08193 Bellaterra (Cerdanyola del Vallès), Spain; 4 OIE Collaborating Centre for the Research and Control of Emerging and Re-Emerging Swine Diseases in Europe (IRTA-CReSA), 08193 Bellaterra, Barcelona, Spain

**Keywords:** *Streptococcus suis*, Co-infections in pigs, Mixed infections, Porcine reproductive and respiratory syndrome virus, Swine influenza virus, Porcine circovirus 2, Bacterial mixed and co-infections, Porcine respiratory disease complex

## Abstract

*Streptococcus suis* is one of the most important bacterial swine pathogens affecting post-weaned piglets, causing mainly meningitis, arthritis and sudden death. It not only results in severe economic losses but also raises concerns over animal welfare and antimicrobial resistance and remains an important zoonotic agent in some countries. The definition and diagnosis of *S. suis*-associated diseases can be complex. Should *S. suis* be considered a primary or secondary pathogen? The situation is further complicated when referring to respiratory disease, since the pathogen has historically been considered as a secondary pathogen within the porcine respiratory disease complex (PRDC). Is *S. suis* a respiratory or strictly systemic pathogen? *S. suis* is a normal inhabitant of the upper respiratory tract, and the presence of potentially virulent strains alone does not guarantee the appearance of clinical signs. Within this unclear context, it has been largely proposed that co-infection with some viral and bacterial pathogens can significantly influence the severity of *S. suis*-associated diseases and may be the key to understanding how the infection behaves in the field. In this review, we critically addressed studies reporting an epidemiological link (mixed infections or presence of more than one pathogen at the same time), as well as in vitro and in vivo studies of co-infection of *S. suis* with other pathogens and discussed their limitations and possibilities for improvement and proposed recommendations for future studies.

## Introduction

*Streptococcus suis* is an important bacterial pathogen of swine with a worldwide distribution [1]. The most common clinical and pathological outcomes of the infection are meningitis, arthritis, endocarditis, septicemia and sudden death [2]. The definition of *Streptococcus suis*-associated diseases should be based on the combination of the presence of clinical signs, gross and/or microscopic lesions and bacterial isolation (in either predominance or pure culture) in affected organs and/or tissues. However, even after bacterial isolation, interpretation of the role of *S. suis* as a primary pathogen is not always easy. Furthermore, since diagnosis in the field may sometimes be based solely on clinical observations, differentiation with infections caused by other pathogens (such as *Glaesserella parasuis*) is difficult. The more specific role of *S. suis* in pneumonia is also a topic of debate. Clinical signs and mortality are mainly observed in weaned and (rarely) in suckling piglets and much less commonly in grower-finisher pigs. The infection caused by the pathogen not only results in severe economic losses but also raises animal welfare concerns. In addition, *S. suis*-associated diseases are difficult to control [2]. Even when the carrier rate of *S. suis* is high, the incidence of the disease varies from period to period and is usually less than 5% [2]. However, this is usually the case when antimicrobials (if allowed) are used as prophylactic/metaphylactic measures. One of the main problems is that antimicrobials that have efficacy are those the industry is trying to reduce given their importance in both human and veterinary medicine [2]. Recent data on antimicrobial susceptibility of *S. suis* are alarming. High rates of resistance to macrolides/lincosamides and tetracyclines are observed and attributed to the intensive use of antimicrobials in pigs [3]. *S. suis* is considered a niche for antimicrobial resistance and represents a high risk of transmission of such resistance to other veterinary and human pathogens due to the presence of mobile genetic elements carrying resistance genes transferable at high frequency within the species and, even more alarmingly, toward other bacterial species [3, 4]. *S. suis* is also considered an important zoonotic infection. The target populations are mainly workers in the swine and pork industry (Western countries), the general population due to close contact with pigs (China and other Asian countries) and individuals who consume raw or undercooked pork or pork by-products (Vietnam, Thailand and Laos) [5, 6].

*S. suis* had originally been classified into 35 serotypes based on the antigenicity of the capsular polysaccharide, which is suggested to be a major virulent factor [7]. However, six of the serotypes (serotypes 20, 22, 26, 32, 33 and 34) have been reclassified as belonging to other bacterial species [8, 9]. The distribution of serotypes recovered from diseased pigs in different geographical regions varies, although serotypes 2 and 9 are the most prevalent in several European countries [1]. Serotype 2 is, by far, the most common serotype affecting humans, followed by serotype 14 [1]. The distribution of serotypes affecting pigs in North America is different, with no clear prevalence of serotype 2 [10, 11]. Highly, intermediately and low virulent serotype 2 strains have been characterized [7, 12]. Further studies using multilocus sequence typing (MLST) showed that sequence type (ST) 1 strains (with other clonal complex 1 strains) normally found in Europe and Asia present higher virulence potential and are mainly isolated from diseased pigs and humans [6].

*S. suis* is normally present in the tonsils and nasopharynx of most healthy pigs [13, 14] and some authors therefore classify the bacterium as “pathobionts” [15]. The conditions under which certain serotypes/strains of *S. suis* become pathogenic and cross the mucosal barrier into the blood causing a systemic infection are not fully understood [16]. A plethora of potential virulent factors has been described, although there is still a debate surrounding their significance to the pathogenesis of diseases linked to *S. suis* infection [7]. It is believed that, under some circumstances, *S. suis* does not act alone and takes advantage of concomitant or previous infections with other pathogens. Indeed, since co-infections have been largely been associated with the increase in clinical disease caused by *S. suis*, we will critically discuss the data available in the literature that directly or indirectly address the issue. The definitions of co-infection, super-infection and mixed infections are not always clear but were plainly explained in a recent review [17]. We will use the term *mixed infection* when there is an epidemiological link only (detection of more than one pathogen in organs/tissues) and *co-infection* for in vitro and in vivo studies that specifically address the interaction between *S. suis* and other pathogens.

## Complexity of the transition from infection (or colonization) to clinical disease caused by *S. suis*

**Figure 1:**
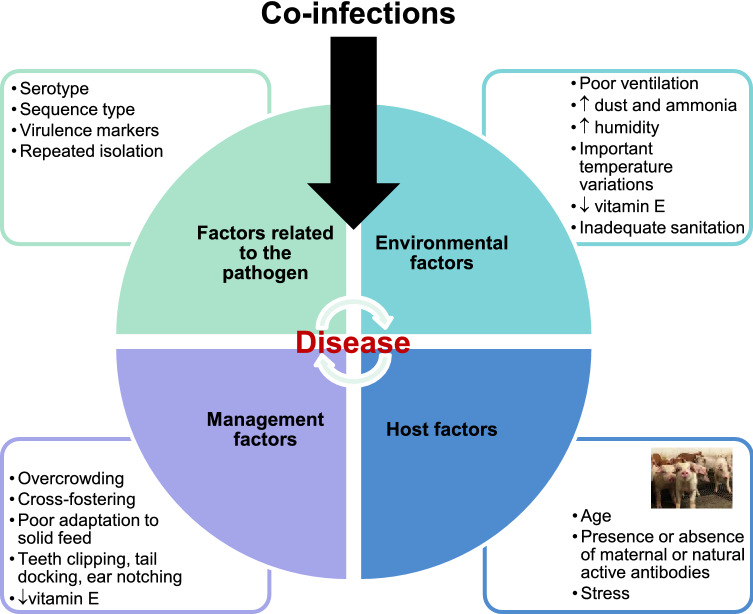
**Factors influencing the appearance of clinical signs of** ***Streptococcus suis*** **infection.**

Should *S. suis* be considered a primary or secondary/opportunistic pathogen? As mentioned, *S. suis* is a normal inhabitant of the upper respiratory tract [2]. The presence of potentially virulent strains alone does not guarantee the appearance of clinical signs, the latter observed sometimes in the absence of such strains, as occurs in North America where highly virulent Eurasian strains are seldom isolated [2]. Indeed, high doses of inoculum (mostly with virulent serotype 2 strains) and aggressive non-natural inoculation routes (intraperitoneal or intravenous) or sometimes two simultaneous routes of infection (intranasal and intramuscular) have been used in experimental trials using conventional pigs to reproduce clinical disease [18–21]. On the other hand, mortalities up to 20% with non-serotype 2 strains may be observed in the natural infection in the field if no medication is used [22]. As mentioned, the definition of virulence for a given strain of *S. suis* is not simple, a fact that is even truer for serotypes other than serotype 2, which have been less studied and for which animal models to reproduce the disease are almost inexistent. For example, serotype 9 virulent strains must be administrated intravenously to susceptible pigs to reproduce disease [23], even though this serotype is the most prevalent among diseased pigs in several European countries [1]. The situation in North America is even more complicated since serotypes 2 and 9 strains are very different from predominant virulent strains in Europe and display lower virulence [2, 24]. Indeed, serotype 1/2, a poorly studied non-zoonotic serotype, is the predominant serotype recovered from clinical cases in the USA and Canada [10 and unpublished data]. It has been proposed that, in addition to the potential virulence of the strain(s) present in the herd, several factors may influence the appearance of clinical signs (Figure 1): (a) environmental factors; (b) management factors; (c) host factors and/or (d) the presence of co-infections. Environmental factors that may influence the appearance of *S. suis*-related diseases include poor ventilation, high humidity, inadequate sanitation, high levels of dust and ammonia and large temperature variations between night and day [2]. Management factors such as high level of cross-fostering, overcrowding, teeth clipping and tail docking, ear notching, mixing pigs of different ages, poor adaptation to solid feed in the nursery and low levels of vitamin E have also been cited as playing important roles [2]. Host factors such as high levels of stress and the presence/absence of anti-*S. suis* antibodies may also influence the appearance of clinical disease. Indeed, it has been shown that clinical signs appear when the level of maternal antibodies is low [22, 25]; antibodies slowly increase at the end of the post-weaning period, and animals become more resistant to infection [26]. These antibodies would not necessarily be all specific to the virulent strains of *S. suis* present in the herd (unpublished observations) but probably also to other *S. suis* or even other streptococci that are normally present in tonsils [25]. Finally, co-infections have been suggested to play a major role in the development of *S. suis* disease. Indeed, co-infections/mixed infections of different swine pathogens with *S. suis* have been reported and suggested in the literature, although there is still a certain lack of scientific support for many of them. This is the main reason why we address the topic in this review.

## Should *S. suis* be considered a respiratory or systemic pathogen? Or both?

Although many studies addressed the role of *S. suis* in respiratory diseases, a pathogen’s route of entry should be differentiated from the induced pathology. Although there is a proposed hypothesis of intestinal translocation (still to be confirmed) [27], it is widely accepted that the main route of infection for systemic *S. suis* disease is the respiratory route [2], and airborne transmission of the infection has clearly been demonstrated [28]. Within this context, it may be argued whether *S. suis* should be considered as a true respiratory pathogen. Infectious agents involved in porcine respiratory disease complex (PRDC) are classified into primary or secondary (or opportunistic) pathogens [17]. Primary pathogens are defined as the ones that can infect the animal as the first unique pathogen and then facilitate secondary or opportunistic co-infection(s). *S. suis* is usually considered as a secondary/opportunistic pathogen [17]. However, the role of *S. suis* in respiratory pathology, even as an opportunistic agent, is still not clear. Studies based on natural infection cases indicate that *S. suis* may cause pulmonary lesions generally described as suppurative bronchopneumonia and/or fibrino-hemorrhagic pleuropneumonia [29–31]. However, in most cases of natural infections, other concomitant bacteria were also isolated, and the specific role of *S. suis* is difficult to evaluate [32]. Respiratory clinical signs are usually not observed after experimental infection with *S. suis*, and the clinical outcomes are mainly septicemia, meningitis and/or arthritis, which are also observed in studied cases of airborne transmission [28, 33]. The few cases in which respiratory signs were observed are frequently associated with heart lesions (endocarditis or pericarditis) with no pulmonary involvement (unpublished observations). Intranasal experimental infections may, under some circumstances (previous irritation of nasal mucosa), induce mostly meningitis and arthritis with no lung lesions associated with respiratory problems ([33] and unpublished data). Intranasal infection of highly susceptible caesarian-derived, colostrum-deprived piglets also led to systemic disease [34]. More recent co-infection studies in conventional pigs (see below) showed some lung lesions following *S. suis* infection, although no indication of the presence of other opportunistic bacterial pathogens in these studies is discussed [20]. Indeed, when evaluating isolates of *S. suis* for its potential virulence (pathotypes), those recovered from lungs are either not included in the study or often considered as possibly opportunistic [10, 35]. *S. suis* may also reach lungs as post-mortem invasion, and that is one of the causes that disqualify the use of isolates recovered from lungs to produce autogenous vaccines [26].

Finally, *S. suis* recovered from the lungs of young animals with respiratory signs and pulmonary lesions should be differentiated from those recovered from lungs at slaughter. *S. suis* rarely induce disease in finisher animals due to the high levels of antibodies present in older animals [25], and the detection of the pathogen in such mixed infections at slaughter should not be taken into consideration. Indeed, *S. suis* may be isolated from healthy lungs without lesions, probably due to the fact that some carrier isolates may go from the upper to the lower respiratory tract during agony [2, 36].

## *S. suis* and viral mixed and/or co-infections

Mixed infections of *S. suis* with swine viruses are a frequent finding in swine herds. These interactions are believed to have a synergistic effect, resulting in the aggravation of clinical signs and increased mortality. However, few controlled studies on real co-infections have been conducted. The most important viral pathogens that have been suggested to be associated with *S. suis* disease are porcine reproductive and respiratory syndrome virus (PRRSV) and, to a lesser extent, swine influenza virus (SIV) and porcine circovirus 2 (PCV-2). Studies that consider other viruses are rare. To better understand the importance of *S. suis* co-infections with swine viruses, we will discuss the main findings from the reported mixed infections, as well as controlled in vitro and in vivo assays that may help elucidate the interactions. The important details and conditions of in vitro and in vivo experimental studies with *S. suis* and viruses are summarized in Tables 1 and 2.

**Table 1:** **Summary of the experimental conditions of** ***S. suis*** **co-infection with viral pathogens using in vitro models**

*S. suis* co-infection pathogen	Type of cells	Virus strain	*S. suis* serotype 2 strain	Virus/bacterial infection dose	Incubation time (hours)		Goal of the study	Main observed effects of co-infection	Refs.
					Virus	*S. suis*			
PRRSV	Swine bone marrow dendritic cells (BMDC) and monocytes	Genotype 2	ST 1	0.1 MOI/1 MOI	2	0.5 to 5; 12 for gene expression	Phagocytosis/ intracellular survival; gene expression (microarray)	**↓** Bacterial phagocytosis (BMDC); **↑** gene expression coding for pro-inflammatory mediators	[56]
	Porcine alveolar macrophages	Genotype 2	ST 1	0.1 MOI/10 MOI		1 to 8	Phagocytosis/intracellular survival; inflammatory-related gene expression (qRT-PCR)	**↑** Bacterial phagocytosis; **↑** intracellular survival and **↑** gene expression for pro-inflammatory mediators	[20]
SIV	Neonatal tracheal porcine cell line (NTPr)	H1N1	ST 1	1 MOI/10 MOI	12	2 and 4	Adhesion and invasion; inflammatory-related gene expression (qRT-PCR)	**↑** Bacterial adhesion and invasion; **↑** gene expression for pro-inflammatory mediators	[79]
	NTPr	H1N1	ST 1	1 MOI/10 MOI	12	12	Gene expression (microarray)	**↑** Gene expression for pro-inflammatory mediators	[85]
	Porcine precision-cut lung slice	H1N1 and H3N2	ST 1	10^5^ TCID_50_/10^7^ CFU	1	24 to 72	Adhesion, invasion, and colonization	**↑** Bacterial adherence/colonization/invasion	[81]
	Porcine bronchial epithelial cells (PBEC) under air–liquid interface	H3N2	ST 1	5 × 10^4^ TCID_50_/2.5 × 10^7^ CFU	24	24 and 48	Adhesion, invasion, and colonization	**↑** Bacterial adherence/colonization/invasion	[83]
PCV-2	Immortalized swine tracheal epithelial cells (STEC) in transwell chambers	PCV-2 Chinese strain	ST 7	1 MOI/10 MOI	24 and 48	2	Adhesion, invasion and bacterial translocation	**↑** Permeability and bacterial translocation through tracheal epithelium	[99]
	STEC	PCV-2 Chinese strain	ST 7	1 MOI/30 MOI	24 and 48	1 to 4	Intracellular survival	**↑** Bacterial intracellular survival; **↓** gene expression for pro-inflammatory mediators	[100]
	Porcine monocytic 3D4/21 cell line	PCV-2 Chinese strain	ST 7	1 MOI/10 MOI	24 and 48	1 and 8	Inflammatory-related gene expression (qRT-PCR)	**↑** Gene expression for pro-inflammatory mediators; **↓** MHC-II expression	[21]

PRSSV: Porcine Reproductive and Respiratory Syndrome Virus; SIV: Swine Influenza Virus; PCV-2: Porcine Circovirus type 2; TCID_50_: medium tissue culture infectious dose; CFU: colony-forming units; ST: sequence type.

### *S. suis* and PRRSV mixed and co-infections

For over 30 years, PRRSV has been one of the leading viral diseases in pigs, causing significant losses in the swine industry, causing more than $600 million in losses every year in the USA alone [37]. The clinical signs can range from reproductive failure in pregnant sows to severe respiratory clinical signs in weaned and growing pigs. PRRSV strains have considerable genetic variability (approximately 60%) and are categorized as PRRSV1 (European genotype) and PRRSV2 (North American genotype) [38]. PRRSV can cause severe disease alone or act in concert with other viruses or bacteria to significantly contribute to PRDC [40–42]. It has been reported that PRRSV infects pulmonary interstitial, alveolar and intravascular macrophages with consequent decreased phagocytic activity and altered innate immune response in the respiratory tract, opening the door to other opportunistic viral and bacterial pathogens [43]. However, there are still some gaps with regard to the specific cell types the virus infects [37].

#### PRRSV-*S. suis* mixed infections

There is strong unwritten evidence of a clear synergic association between PRRSV and *S. suis* from the clinical perspective, and most swine practitioners strongly believe that positive unstable (Category I) herds [44] are more prone to serious problems of *S. suis* disease than PRRSV stable or free herds. Some examples in the literature describe the simultaneous detection of both pathogens in the same farm (mixed infections). Although PRRSV seroprevalence is a good indicator of swine herd exposure to the virus, RT-PCR analysis is required to detect active infections and confirm mixed infections of PRRSV with other swine pathogens [45]. For example, RT-PCR analysis of oral fluids from pig farms in Korea showed that PRRSV and *S. suis* were frequently detected together (around 50%) in 3 to 7 week-old piglets [46]. Studies performed at slaughter in Canada showed that RT-PCR analysis of swine pathogens present on tonsils detected *S. suis* and PRRSV in 53.7% and 22% of samples, respectively [13]. Similarly, another study at slaughter showed that lungs that were RT-PCR positive for PRRSV have significantly higher odds of being positive for *S. suis* [47]. These data (from both abattoirs and non-clinical samples) do not reveal the nature of the pathogens’ interaction and their possible synergistic effect due to the endemic characteristics of PRRSV and ubiquitous presence of *S. suis*. Indeed, it is important to state that both pathogens may be present in herds without any associated clinical signs of disease.

In Vietnam, Hoa et al. showed an increased isolation rate of highly virulent *S. suis* serotype 2 in the blood and internal tissues of diseased pigs from PRRSV-affected farms (18%) compared to those that were not affected by the virus infection (2%) [48]. Interestingly, a temporal and spatial association of occurrence of human meningitis caused by *S. suis* and PRRSV outbreaks in pig farms was observed in that country [49]. It should be noted that the study addresses association and not causality. The study also has limitations (clearly stated by the authors): incomplete details on PRRSV outbreaks, differences in sample collection, underestimation of the real prevalence and distribution of PRRSV outbreaks and lack of details on individual patient data that may constitute confounding factors.

The few epidemiological studies and clinical observations by practitioners around the world point to the importance of the *S. suis*-PRRSV association with regard to the occurrence of severe disease in pigs. However, the mechanisms behind the observed synergistic effect of both pathogens have yet to be explained. To better understand the cellular, immunological and molecular implications behind this synergy, some in vitro and in vivo models have been developed.

#### In vitro studies on the interactions between *S. suis* and PRRSV

Owing to the characteristics of PRRSV, *S. suis* co-infection studies mainly addressed the interactions with cells of the innate immune system. Although the pathogenesis of the infection caused by *S. suis* is not yet completely understood [7], it is believed that its interaction with respiratory phagocytic cells may be one of the initial steps of the infection [16]. In this first line of defense, pulmonary alveolar macrophages (PAMs) play an essential role in the innate immune response against pathogens through bacterial phagocytosis and elimination [42]. Since these cells are also among PRRSV’s most important targets, they may represent an interesting model to study co-infection effects [43]. The hypothesis behind the PAM cells’ use in co-infection studies is that a prior PRRSV infection would reduce *S. suis* phagocytosis either by reducing phagocyte activity or inducing cell apoptosis. However, under normal conditions, *S. suis* possesses a thick capsule that usually prevents bacterial phagocytosis—a fact confirmed by different research groups using non-encapsulated mutants [7]. It is important to note that the bacteria that are internalized do not survive intracellularly [50]. So, it may also be hypothesized that although the internalization of *S. suis* is a rare event, a previous viral infection may affect the intracellular killing of bacteria, which may influence the outcome of the disease. Another important aspect of the interaction of *S. suis* with phagocytes is the capacity to induce inflammation. Indeed, it has been shown that an excess of inflammation is a hallmark of *S. suis* disease [7]. Hence, two pathogens acting together would increase the inflammatory reaction through an increase in the secretion of pro-inflammatory cytokines. Still, immunosuppression has been demonstrated in some PRRSV infection studies and thus may lead to a higher susceptibility to bacterial co-infection in affected pigs [51, 52]. Interestingly, the two processes are not necessarily in contradiction, since the dual activity of PRRSV (immunosuppression but increased inflammation) has been observed in cases of PRRSV and porcine respiratory coronavirus co-infection studies in pigs [53].

Several studies addressed the interaction between *S. suis* and PAMs [50, 54, 55], but only one reported how a previous PRRSV infection of these cells affects *S. suis* phagocytosis and inflammation (Table 1) [20]. PAMs infected with a highly pathogenic PRRSV genotype 2 strain and a poorly characterized *S. suis* serotype 2 strain showed increased bacterial phagocytosis and survival [20]. In addition, co-infection significantly increased the mRNA expression of most pro-inflammatory cytokines tested, including interleukin (IL)-1β, IL-6, IL-8, chemokine (C–C motif) ligand 4 (CCL4), tumor necrosis factor-α (TNF-α) and interferon-β (INF-β). Authors concluded that the deleterious effect of the co-infection was mainly due to excessive inflammation [20]. Surprisingly, no other study addressed the effect of co-infection on these cells.

Another study addressed the effect of co-infection using monocytes and swine bone marrow-derived dendritic cells (BMDC) [56]. Both cell types were first infected with a PRRSV genotype 2 strain and then co-infected with the European (ST1) virulent serotype 2 P1/7 strain of *S. suis* (used in virulence studies by most laboratories) [56]. Unlike the previous study with PAMs, results showed that PRRSV significantly reduced the internalization of *S. suis* by the BMDC with no observed effect on bacterial intracellular survival [20]. No differences were observed with monocytes, which hardly allowed virus replication and poorly phagocytosed *S. suis* [56]. As shown with PAMs, microarray analysis revealed significant up-regulation of pro-inflammatory genes in co-infected BMDC [56]. Previous *S. suis* research showed the capacity of the bacteria alone to induce the secretion of pro-inflammatory cytokines involved in disease pathogenesis [57]. Co-infection with PRRSV may exacerbate the inflammation by increasing the secretion of cytokines from immune cells, although this hypothesis should be confirmed in vivo. Unfortunately, there are no other studies of co-infection of these two important pathogens with phagocytic cells. Indeed, it has been shown that pulmonary intravascular macrophages may play an important role in PRRSV infection [58]. Although a possible association between a virus-dependent suppression of the functions of these cells and an increased susceptibility to *S. suis* secondary infection has been hypothesized, no study has specifically addressed this interaction. However, an interesting feature of PRRSV is that it may affect the thymus and its ability to carry out its normal functions [52, 59]. In this way, pigs would be less able to resist and/or eliminate secondary infections. It has also been reported that *S. suis* infection can cause atrophy of the thymus and induce apoptosis of thymocytes, thus likely suppressing host immunity [60]. How these two pathogens interact with this important immune organ is still unknown.

Finally, co-infection studies with non-immune cells have not been conducted. Since PRRSV induces nonsuppurative rhinitis and metaplasia of the turbinate epithelium, it has been suggested that it may predispose pigs to the colonization of the respiratory tract by *S. suis* serotype 2, potentially creating a portal of entry for *S. suis* [61]. However, the hypothesis of virus predisposition to bacterial colonization has never been confirmed.

In summary, although it is widely accepted that a PRRSV infection increases the susceptibility to *S. suis* co-infection (see results in vivo below), it seems evident that there is a serious lack of scientific evidence that clearly explains the specific mechanisms involved in such interactions and more mechanistic studies are needed.

#### In vivo studies on the interactions between *S. suis* and PRRSV

Although a first report failed to demonstrate any influence of PRRSV infection on an *S. suis* secondary infection [62], further in vivo experiments were carried out to demonstrate the synergistic effect of these pathogens on morbidity and the severity of clinical signs in pigs [20, 52, 61, 63, 64]. The most common type of co-infection model was developed by infecting animals intranasally with PRRSV and then, after 5 to 7 days, inoculating *S. suis* intranasally or intramuscularly (an overview of the important study parameters is shown in Table 2) [20, 61, 63, 65]. Only one study addressed an intrauterine infection with PRRSV (sows) followed by *S. suis* infection in piglets [52] (see below). Different PRRSV types (low to high virulent) and origins (North America or Asia), as well as *S. suis* serotype 2 (sometimes poorly characterized) strains, were assayed. There is only one study that used a *S. suis* serotype 7 strain [65]. The consensus of these studies is that higher mortality and morbidity were observed in groups in which piglets were co-infected by the two pathogens compared to those infected only with *S. suis*, independently of the virus genotype and *S. suis* serotype [20, 61, 65]. *S. suis* was cultured in higher numbers from tissues and blood (bacteremia) of co-infected animals compared to *S. suis* mono-infected piglets and macroscopic and microscopic lesions in different internal organs were significantly exacerbated in animals in the co-infected groups [20, 61, 65]. In particular, co-infected piglets had significantly more severe gross and microscopic interstitial pneumonia lesions, suggesting a possible secondary role of *S. suis* in PRDC [20, 42, 65]. The co-infection was able to reproduce similar morbidity and mortality to those observed in the high-fever outbreak caused by atypical PRRSV in China [39]. Interestingly, a vaccine strain of PRRSV given intranasally prior to *S. suis* infection induced increased mortality compared to the single-infected controls, although the mortality was lower than in the cases of co-infection with a virulent PRRSV strain [61]. It must be noted that this was an off-label use of the PRRSV live attenuated vaccine, and it is unlikely that the vaccine would be administered intranasally under field conditions.

**Table 2:** **Summary of the experimental conditions of** **S. suis** **co-infection with viral pathogens using in vivo models**

*S. suis* co-infection parhogen	Age of pigs at the infection Virus / Bacteria		Route of infection Virus / Bacteria		Virus strain and dose	*S. suis* strain and dose	Main observed effects of co-infection	Ref
PRRSV	19 days	26 days	IN	IN	2 × 10^4.99^ TCID_50_, high virulent PRRSV strain, genotype 2 2 × 10^4.47^ TCID_50_, low virulent PRRSV vaccine strain RespPRRS/Repro™	2 × 10^8.3^ CFU; serotype 2 (no further details on the strain)	**↑** Severity of clinical signs, mortality, and interstitial pneumonia lesions (especially those infected with the high virulent PRRSV strain)	[61]
	98 days of sow gestation	5 days	IN	IN	10^3.5^ TCID_50_, PRRSV, genotype 2	8 × 10^7^ CFU, serotype 2 (no further details on the strain)	**↑** Susceptibility of piglets to bacterial infection	[52]
	21 days	28 days	IN/IM	IN/IM	2 × 10^4.97^ TCID_50_ PRRSV, genotype 2	10^9^ CFU; serotype 7 (no further details on the strain)	**↑** Clinical signs and mortality	[65]
	49 days	42 days*	IN	IN	2 × 10^4^ TCID_50_ Asian HP-PRRSV strains; US PRRSV strains, genotype 2	10^6^ CFU; serotype 2 (no further details on the strain)	**↑** Clinical severity related to PRRSV virulence; **↓** levels of proinflammatory cytokines	[64]
	28 days	21 days*	IN	IM	10^6^ TCID_50_ PRRSV MLV-like strain	2 × 10^8^ CFU; serotype 2 low virulence strain (no further details on the strain)	**↑** Clinical signs and mortality	[67]
SIV	35 days	38 days	IN	IN	2 × 10^7^ egg infectious dose_50_/mL, H1N1 strain	2 × 10^6^ CFU; serotype 2; ST7	**↑** Clinical signs, pathological changes, and cell apoptosis; **↑** gene expression for pro-inflammatory mediators	[76]
PCV-2	28 days	33 days	IN/IM 2 mL/3 mL	IN/IM 2 mL/3 mL	2 × 10^6.5^ TCID_50_	4.5 × 10^9^ CFU; serotype 2; ST7	**↑** Clinical signs; **↑** gene expression for pro-inflammatory mediators; **↓** gene expression for CD4, CD8, and MHC-II	[21]
PRV	63 days	63 days	IN	IN	5 × 10^3^ TCID_50_ high or low virulent PRV strains	10^9^ CFU *S. suis* serotype 2 (no further details on the strain)	**↑** Clinical signs	[107]

^*^ Animals were infected first with *S. suis* and then infected with a virus.

IM: intramuscular injection; IN: intranasal inoculation; PRSSV: Porcine Reproductive and Respiratory Syndrome Virus; SIV: Swine Influenza Virus; PCV-2: Porcine Circovirus type 2; PRV: pseudorabies virus; TCID_50_: medium tissue culture infectious dose; CFU: colony-forming units; ST: sequence type.

PRRSV is a causative agent of complex disease, attacking multiple organs. The infection of pregnant sows with PRRSV results in late abortion, early farrowing and stillborn piglets [66]. Piglets from PRRSV infected sows are PRRSV positive and, in theory, would have a weakened immune system that predisposes them to other opportunistic pathogens [66]. One animal model was developed to examine the effect of intrauterine PRRSV infection in piglets on susceptibility to further intranasal infection with *S. suis* serotype 2 [52]. To the authors' knowledge, this is the only published study on adult PRRSV infection followed by *S. suis* piglet co-infection. Pregnant gilts were either inoculated or not with PRRSV at 98 days of gestation (see Table 2) [52]. Five day-old piglets born from these sows were inoculated intranasally with an *S. suis* serotype 2 strain [52]. The mortality rate, clinical signs, organ lesions and *S. suis* isolation from joints and brain in the co-infected group were significantly higher compared to control non-*S. suis* infected piglets and those infected with *S. suis* alone [52]. Increased susceptibility was explained as being linked to the immuno-suppression caused by the effect of PRRSV on innate immune cells. Indeed, PRRSV-positive piglets had lesions in their thymus and bone marrow and significantly reduced numbers of leukocytes, including lymphocytes and monocytes [52]. In conclusion, this study showed that intrauterine PRRSV infection has significant effects on immune response and may increase the susceptibility of piglets to intranasal *S. suis* serotype 2 infections. Even so, the presence of confounding factors, especially concerning the experimental model used, must be considered. Piglets were deprived of sow’s colostrum, early-weaned and challenged with bacteria at five days of life, which is rare in the field, where mostly post-weaned animals are affected by *S. suis* [25, 52].

Other studies addressed the role of a pre-infection with *S. suis* with subsequent PRRSV infection. One of these studies used 6-week-old PRRSV-negative piglets weaned before one week of age and kept free of pathogens. Animals were intranasally inoculated with a bacterial cocktail containing *S. suis* and two other pathogens, mimicking sub-clinical bacterial infections observed in the field. One week later, piglets were challenged with PRRSV strains of different origins and variable virulence [64]. Results showed low mortality in general, although morbidity and lesions were more severe in piglets inoculated with a highly virulent Asian PRRSV strain [64]. The frequency of secondary bacterial pneumonia was directly associated with the clinical severity induced by the PRRSV strains evaluated. Levels of pro-inflammatory cytokines were lower or at the same level in single or co-infected animals, which contradict somehow previous in vitro co-infection studies [20, 56]. Another study used three-week-old conventional piglets that were inoculated intramuscularly with *S. suis* serotype 2 strain and seven days later with a highly pathogenic PRRSV MLV-like isolate [67]. Results showed that while virus or *S. suis* infection caused transitional fever and moderate clinical signs, the co-infection induced higher fever, anorexia and respiratory distress, leading to 60% mortality [67]. It should be noted that the route of animal infection with *S. suis* does not reflect natural conditions, and the results should be interpreted carefully.

The synergy between PRRSV and *S. suis* in vivo cannot be denied, and experimental infection studies generally confirm this fact. However, there are still many variables in previous co-infection studies to identify the mechanisms involved. The pathogenicity of the PRRSV strain is just one part of the puzzle, while other factors like stress, environment, host susceptibility and bacterial burden are some of the variables that may influence the clinical and pathological outcomes. One important point is also the strain of *S. suis* serotype 2 used: in many studies, strains are poorly characterized although it is well known that the virulence of serotype 2 strains is highly variable [7]. One study showed either no effect of co-infection using one *S. suis* strain (with a North American phenotype) or a strong synergy when a second and different strain (with a Eurasian phenotype) was used [2, 63]. It is important to note that co-infections of PRRSV and typical North American serotype 2 (ST28 or ST25) *S. suis* strains are frequently reported in Canada and the USA. Even though the virulence of these *S. suis* strains is probably lower than that of Eurasian strains [7], a combination of other factors may have an influence on the effects of the co-infection (Figure 1). Interestingly, other than the study mentioned above, no co-infection trials have been performed with typical North American *S. suis* strains.

### *S. suis* and swine influenza type A virus (SIV) mixed and co-infections

Swine influenza viruses (mainly type A) are able to cause respiratory disease in pigs worldwide, that can lead to 10–15% mortality [68]. Influenza disease in pigs is highly contagious with no evident clinical signs or mild to moderate ones characterized by runny nose and coughing [69]. Swine influenza causes significant economic losses primarily due to weight loss, though some cases may be much more severe in co-infection with other pathogens, such as *M. hyopneumoniae*, PRRSV and *Actinobacillus pleuropneumoniae* [68]. The three main SIV subtypes encountered in pigs are H1N1, H1N2, and H3N2, and since 2009, the pandemic H1N1 (pH1N1) has also been circulating in domestic pigs worldwide [70]. Influenza viruses cause respiratory disease by infecting the epithelial cells leading to cell apoptosis and the destruction of the mucosal barriers [71]. In addition, cell death is enhanced by the effect of cytokines and innate immune cells, which causes bronchitis and interstitial pneumonia [71].

#### SIV-*S. suis* mixed infections

It is well known that influenza virus infections in humans are usually complicated by secondary bacterial infections, especially *Streptococcus pneumoniae* [72]. The capacity of SIV to aggravate respiratory bacterial infections in pigs has also been documented [73–75]. However, the potential of SIV and *S. suis* mixed or co-infections to cause serious pulmonary disease has been much less studied and there are only a few published reports. Serological data indicate a link between SIV and *S. suis* in swine farms in China, although detailed information on the correlation of antibody titers and isolation of these two pathogens from healthy or diseased animals is missing [76]. The report on SIV H1N1 virus infections in pigs in England provided more information, since data show that clinical signs were mostly mild with low mortality in older animals infected with SIV alone. Increased mortality was observed in nursery pigs, in which influenza infection was complicated by environmental stress and/or co-infections, with *S. suis* being the most prevalent pathogen isolated in these cases [77]. *S. suis* serotypes 1, 2, 14, and 24 as well as some untypable isolates were detected in SIV-infected pigs with severe signs of cough, meningitis, lameness and sudden death [77]. These results suggest that SIV may complicate *S. suis* infections observed in the field.

#### In vitro studies on the interaction between *S. suis* and SIV

Different in vitro and ex vivo tissue cell culture models were therefore developed to examine the complex mechanism of host–pathogen interactions during co-infection (Table 1). Most in vitro studies address the influence of a previous SIV infection on secondary *S. suis* infection. However, an earlier study using the Madin-Darby canine kidney (MDCK) cell line model showed that the supernatant from a *S. suis* serotype 2 strain can increase the infection ability of SIV H3N2 for these cells [78]. The results may suggest that *S. suis* has secretory factors that facilitate SIV entrance even in the absence of bacteria. It should be noted that MDCK cells do not resemble swine respiratory mucosal cells and, in this study, the effect of the supernatant was evaluated in a closed environment (a well) in which extracellular factors of *S. suis* are concentrated. The results have not been confirmed or invalidated by other research groups.

The first study that demonstrated the effect of a previous SIV infection in cells followed by a subsequent *S. suis* infection was carried out using neonatal tracheal porcine epithelial (NTPr) cells [79]. SIV H1N1 pre-infected cells enabled bacterial adhesion and invasion levels that were over 100 times higher compared to those of control cells. Inhibition studies confirmed that bacterial capsular sialic acid moiety is responsible for the binding to the viral hemagglutinin expressed on the NTPr cell surface [79]. Also, pre-incubation of *S. suis* with SIV H1N1 significantly increased bacterial adhesion to epithelial cells and epithelial cell invasion. Similar results were obtained with other *S. suis* sialic-acid positive serotypes (such as serotypes 1 and 14), but not with serotypes that lack the presence of such sugar moiety in their capsular polysaccharides [79]. The results were confirmed by an independent research group using the same cell type and both SIV H1N1 and H3N2 subtypes [80]. The latter study also revealed that viruses bound to bacteria retained infectivity but induced only tiny plaques compared to the control virus. In contrast, bacterial co-infection had a negative effect on SIV replication. The real cause of this effect is unknown, but the authors hypothesized that the reduction in the amount of released virus may be caused by the direct binding of virions to bacteria and/or a detrimental effect caused by bacteria bound to the surface of infected cells [80]. Further studies are needed to elucidate this effect.

The presence of differentiated epithelial cells and mucus in animal respiratory mucosa creates additional complexity that could not be replicated with epithelial cell lines. To overcome this limitation, Meng et al., established a porcine precision-cut lung slice (PCLS) co-infection model [81]. PCLSs use a procedure that preserves multicellular tissue consisting of ciliated cells, mucus-producing cells and pneumocytes [81]. Hence, the presence of differentiated epithelial cells with preserved functions in this model made it possible to study *S. suis* interactions in an environment that more closely resembles an in vivo respiratory mucosal surface [81]. PLCSs were pre-infected with SIV subtypes H1N1 or H2N3 and subsequently co-infected with an *S. suis* serotype 2 strain [81]. As shown in previous studies [79, 80], the adhesion of *S. suis* to epithelial cells was significantly increased by SIV in a bacterial capsule-dependant manner, especially in the early stage [81]. However, at a later stage of co-infection, results were virus strain-dependent, since only SIV H3N2 induced the increased attachment of not only the encapsulated *S. suis* but also of its non-encapsulated mutant [81]. Indeed, confocal microscopy of cryosections showed the destruction of ciliated epithelial cells and an increased presence of both encapsulated and non-encapsulated *S. suis* in the sub-epithelium of PLCSs [81]. These results imply that SIV promotes the bacterial infection of respiratory epithelial cells in two phases, depending on viral subtype. The first phase is at the beginning of the infection, when adherence of bacteria is enhanced by viral hemagglutinin (from both virus subtypes) expressed on the surface of the epithelial cells that bind *S. suis* capsular polysaccharide sialic acid moiety. The second phase occurs later when SIV H3N2 damages the epithelial cells and opens the path for the bacterial colonization of the subepithelial tissue in a capsule (and sialic acid)-independent manner [81].

To better resemble the luminal respiratory environment in airway epithelium, an air–liquid interface (ALI) culture system for differentiated porcine airway epithelial cells was developed [82]. Primary porcine tracheal and bronchial epithelial cells were cultured in a Transwell filter system that enables the differentiation of epithelial cells under air–liquid interface conditions [82]. Indeed, the cell monolayer acquired cilia, pseudostratified epithelium and tight junctions that resemble the cells of airway epithelium in a live animal [82]. The model was first used to evaluate the adhesion and cytotoxic properties of *S. suis* serotype 2, showing that the hemolysin produced by *S. suis* (suilysin) contributes to the loss of ciliated cells and cell apoptosis, as well as bacterial adhesion and invasion (76). The same system was further developed to examine the interactions of SIV H3N2 and *S. suis* serotype 2 [83]. The previous infection of cells with the virus increased *S. suis* sialic acid-dependent adherence and colonization, once more confirming previous studies [79–81]. There was a prominent difference in cytopathogenicity observed during the single infection with the bacteria or SIV in this study. *S. suis* infection resulted in early destruction of the differentiated cells, while SIV H3N2 induced apoptosis in the later stage of infection. It was suggested that *S. suis* cytopathogenicity may be due to the suilysin, since a mutant defective in the production of the toxin adhered but did not damage the cells during the mono-infection. When SIV H3N2 was present in a co-infection study using both wild-type and suilysin-negative mutant strains, the latter was also able to adhere and invade deeper layers of differentiated epithelial cells [83]. Indeed, the authors propose that suilysin-negative *S. suis* strains, which are common in North America [2], can become invasive in a co-infection scenario with influenza A viruses.

The role of inflammation through an increase of the expression of pro-inflammatory mediators has been described for both *S. suis* and SIV [84]. A preliminary study showed that co-infection significantly increased the expression of proinflammatory genes [79]. A more detailed study on gene expression in NTPr cells during *S. suis* and SIV H1N1 co-infection using a microarray assay confirmed that the infection of cells with SIV H1N1 alone or co-infection with both pathogens induced higher mRNA expression of genes in different biological categories than cells infected by *S. suis* alone, with genes involved in immune response and inflammation being particularly overexpressed [85]. This synergy may be the consequence, at least in part, of an increased bacterial adhesion/invasion of epithelial cells previously infected by SIV.

#### In vivo studies on the interaction between *S. suis* and SIV

Because *S. suis* and SIV co-infection became of interest just recently, there is only one published animal model (Table 2). Lin et al. developed an in vivo model in which five-week-old piglets were intranasally infected with SIV H1N1 and then intranasally co-infected with a (suilysin positive) *S. suis* serotype 2 strain three days later [76]. The co-infected group showed more severe clinical signs and viral-induced pneumonia compared to the virus or *S. suis* single-infected groups [76]. The results confirmed, at least in part, the clinical and epidemiological field data from England in which clinical signs seemed to be aggravated in co-infected animals [77]. Interestingly, the viral loads in the lungs were significantly higher in the co-infected group compared to the control group infected with either bacteria or SIV H1N1 alone, thus contradicting previous in vitro observations on a possible inhibitory role of *S. suis* on virus replication [80]. Moreover, the bacterial load was not increased in the co-infected group compared to the *S. suis*-infected group [76]. Results also showed significant upregulation changes in genes involved in pro-inflammatory (TLR4, MyD88, IL-17D, IL-6, IL-8 and CCL2) and apoptosis (CASP2, CASP3, BCL2L11, FASLG and TNFRSF8) in the co-infected group compared to the control mono-infected groups [76]. These results confirm previous observations in vitro using tracheal epithelial cells [79]. In sum, the study results confirm a synergic clinical and pathological effect of SIV H1N1 and *S. suis* serotype 2 in pigs*.* However, from the mechanistic perspective, this study did not characterize the immune cells involved in the generation of mucosal and systemic immune responses.

The characterization of molecular and immunological aspects of co-infections in pigs may also contribute to a better understanding of disease in human medicine [86]. Indeed, as mentioned, human influenza disease is often complicated by secondary pneumonia caused by *S. pneumoniae*, which leads to significant morbidity and mortality around the world [72]. However, the mechanisms involved in this co-infection are also partially known [72].

### *S. suis* and porcine circovirus 2 mixed and co-infections

Porcine circoviruses (PCVs) single-stranded DNA viruses that belong to the genus *Circovirus* under the family *Circoviridae* [87]*.* Four types of PCVs have been described: PCV-1, PCV-2, PCV-3 and PCV-4 [88]. PCV-2 is, by far, the most important PCV for the swine industry and is found to be associated with multiple clinical manifestations of the disease in pigs referred to as porcine circovirus diseases (PCVD) in Europe and as porcine circovirus associated disease (PCVAD) in North America [89, 90]. The clinical signs of PCVD/PCVAD were first described as a postweaning multisystemic wasting syndrome (PMWS, currently known as PCV-2-systemic disease, PCV-2-SD) in Canada [91]. PCV-2 was also associated with the porcine dermatitis and nephropathy syndrome and with enteritis [92–94]. However, the most prevalent form detected in the field is as PCV-2 subclinical infections [95].

#### PCV-2-*S. suis* mixed infections

An analysis of 484 field cases of PCV-2-SD in the USA revealed that most cases were in association with other pathogens and only 1.9% of investigated cases were caused by PCV-2 alone [96]. PCV-2 was most often detected with PRRSV (51.9%) and *M. hyopneumoniae* (35.5%) [96]. Bacterial septicemia and bacterial pneumonia were detected in 14% and 7.6% of the PCV-2-SD cases, respectively [96]. The most prevalent bacteria detected in bacterial septicemia cases was *S. suis*. A similar 2009 study on PCV-2 mixed infections from the same region yielded similar results, with *S. suis* found in around 35% of PCV-2-SD cases with bacterial septicemia [97]. Furthermore, a qPCR assay was applied in a retrospective epidemiological survey of 121 PCV-2-SD cases from Canadian farms to examine the association between PCV-2 and other swine pathogens [98]. The study revealed that high PCV-2 loads increased the odds ratio of isolating *A. pleuropneumoniae* and *S. suis* serotypes 1/2, 1, 2, 3, 4 and 7 from respiratory samples [98], but no clear correlation with disease could be established. Another study detected the presence of both pathogens by qPCR in oral fluid samples [46] but, similarly, no association with the disease was observed. However, the percentage of PCV-2 PCR positive results increased with age, whereas positive results for *S. suis* showed the opposite, indicating that perhaps both pathogens are not necessarily prevalent at the same time [46]. Taken together, although epidemiological results might suggest a possible role of *S. suis* as a complication of PCV-2-associated diseases, no clear data confirm this hypothesis.

#### In vitro studies on the interaction between *S. suis* and PCV-2

Only three in vitro co-infection studies have been performed using different cell types to study *S. suis* and PCV-2 co-infection (Table 1) [21, 99, 100]. The results of the first study, which used poorly characterized immortalized swine tracheal epithelial cells, showed that although no increased adhesion or invasion of *S. suis* serotype 2 was observed, the virus pre-infection decreased the expression levels of ZO-1 and occludin, increased cell permeability and contributed to the translocation of *S. suis* serotype 2 across the tracheal epithelium [99]. Although this in vitro model suggests a possible role of PCV-2 during the initial steps of co-infection by *S. suis*, the level of replication of PCV-2 in tracheal and other respiratory epithelia in pigs is limited, since PCV-2 infects mainly lymphoblast and monocytes (101). Therefore, the impact of the described viral effect in this study is somehow controversial.

In a complementary study by the same research group and using the same cells (with the same limitations), the authors suggested that a previous PCV-2 cell infection would enhance bacterial intracellular survival through a decrease in reactive oxygen species. Moreover, the co-infection of tracheal cells with PCV-2 and *S. suis* would be responsible for a downregulation of the expression of pro-inflammatory cytokines, which would weaken the host’s defensive response [100]. The results, which have yet to be confirmed, must be interpreted carefully since it has been largely reported that inflammation plays a major role in the pathogenesis of PCV-2 infections. Again, these results should be carefully interpreted.

In the third in vitro study, a co-infection of the porcine monocytic cell line 3D4/21 with PCV-2 and *S. suis* serotype 2 induced a significant upregulation of the expression of pro-inflammatory cytokines as well as TLR2, compared to single-infected cells [21]. The upregulation of TLR4 was mainly induced by PCV-2, whereas the downregulation of MHC-II was observed mostly with *S. suis* [21]. Results may indicate a possible role of the co-infection on increased inflammation and the modulation of antigen presentation, although more studies are required to confirm the hypothesis.

#### In vivo studies on the interaction between *S. suis* and PCV-2

There is only one in vivo study using both pathogens (Table 2) [21]. In this model, four-week-old piglets were infected with a PCV-2 strain through intranasal and intramuscular inoculation. Five days later, the animals were intranasally and intramuscularly challenged with a *S. suis* serotype 2 strain. Compared to single-infected pigs, the co-infected group showed higher temperatures and lower weight gains. The amounts of *S. suis* in the internal organs of co-infected animals were inconsistently and slightly higher than those in the *S. suis*-infected group, although the biological significance of the results has yet to be confirmed. Co-infected animals had higher scores of clinical signs and lesions of pneumonia, myocarditis and arthritis compared to single-infected animals [21]. Peripheral blood mononuclear cells collected from co-infected piglets showed higher expression levels of inflammatory cytokines, TLR2 and TLR4 and reduced levels of CD4, CD8 and MHC-II. It was also shown that PCV-2 disrupted the integrity and decreased mRNA and protein levels of tight junctions in the lungs of co-infected animals [99]. Although the authors showed a reduced antibody response against *S. suis* in virus-infected animals, a poorly characterized commercial kit available in China only was used [21]. Currently, there is no widely scientifically accepted test to measure antibodies against *S. suis* [2]. More studies using other strains and different animal models must be conducted to confirm the results.

### *S. suis* and other virus co-infections

The worldwide distribution and high prevalence of *S. suis* in pig herds increase the possible interactions with multiple viruses. Co-infections of *S. suis* with pseudorabies virus (PRV) and Nipah virus have been recorded in the field and experimentally studied in pigs. The common characteristics of *S. suis*, PRV and Nipah virus are that they invade through the respiratory epithelium, have tropism for neurological tissue and are zoonotic pathogens.

PRV, also known as Aujeszky’s disease virus, has a significant economic importance for the pig industry [102]. Infected pigs have respiratory or neurological clinical signs with subsequent high mortality [103]. Pigs are natural hosts, although other domestic and wild animals may also be infected [104], and the disease has zoonotic potential [105]. PRV may be associated with other viral pathogens, such as PCV-2, and may be detected together using PCR in clinically ill pigs and aborted fetuses [106]. It was speculated that due to the prevalence of *S. suis* in pig herds, PRV co-infection can aggravate respiratory and neurological outcomes. In an experimental study, nine-week-old piglets were intranasally inoculated with *S. suis* and a high or low virulent PRV strain (Table 2). Interestingly, both PRV strains induced more severe *S. suis* clinical signs in co-infected piglets [107]. The biological relevance of this type of co-infection is still not clear since there are no recent experimental or epidemiological data evaluating *S. suis* and PRV associations. PRV has been eradicated from many countries and, when present, vaccination has been used to successfully prevent Aujeszky’s disease in pigs. This may partly explain the reason why research into its association with other pathogens is scarce [101].

Nipah virus is a single-stranded non-segmented RNA virus belonging to the family of *Paramyxoviridae* [108, 109]*.* It causes disease in multiple animal species with fruit bats and flying foxes being the main reservoirs [108, 110, 111]. Pigs could be naturally infected, but the infection is often asymptomatic with the virus present in nasal cavities [112]. In experimental studies, young piglets displayed pronounced respiratory clinical signs, while older pigs had predominantly neurological clinical signs [113, 114]. Nipah virus has significant public health impact, since it causes disease in humans, especially in East and Southeast Asia [115], where human infection caused by *S. suis* is also predominant [49]. In the case of experimental oronasal infection in six-week-old piglets with Nipah virus, it was observed that the piglets with aggravated respiratory clinical signs had *S. suis* isolated from the respiratory tissue [116]. This finding should be carefully considered, since bacteria were already present in infected pigs (healthy carriers) and the isolation of *S. suis* was merely accidental [116]. As discussed above, the presence of *S. suis* in the respiratory tissue does not prove the involvement of the bacteria in respiratory disease. Although further studies are required to elucidate the relevance of *S. suis* and Nipah virus co-infection, there are technical and economic obstacles to develop pig experimental co-infection models. The Nipah virus is a biosecurity level 4 pathogen that requires strict measures and special research facilities that are not widely available.

## *S. suis* mixed and/or co-infections with other bacterial pathogens

Described mixed swine bacterial infections concern mainly respiratory pathogens involved in the PRDC, such as *M. hyopneumoniae*, *Mycoplasma hyorhinis, A. pleuropneumoniae*, *Actinobacillus suis*, *Glaesserella parasuis*, *Bordatella bronchiseptica*, *Pasteurella multocida* and *S. suis* [42]*.* As discussed previously, some are primary pathogens (mainly *A. pleuropneumoniae* and *M. hyopneumoniae*), while others are considered secondary pathogens (*S. suis* and *G. parasuis*) [17, 42]. There are very few real co-infection studies of *S. suis* with other bacteria, and most reports address mixed infections.

### *S. suis* mixed infections with other bacterial pathogens

One of the main problems of most studies is that mixed infections are described at the abattoir [32, 117]. For primary respiratory pathogens that induce fairly typical lesions and affect grower-finisher animals (such as *A. pleuropneumoniae*), the studies are appropriate [118]. When available, the combination of serological surveys based on validated antibody detection techniques (for example against *A. pleuropneumoniae* and *M. hyopneumoniae*) at the herd level along with the presence of lesions at slaughter may indicate the pathogens’ involvement in the pulmonary pathology of pigs [119]. However, for a secondary respiratory pathogen such as *S. suis* that mainly affect nursery piglets and for which validated serological tests are not available, the studies are far less relevant. As mentioned above, *S. suis* sometimes colonizes the lungs pre- or ante-mortem without playing any major pathological role in pneumonia [2, 119].

Early studies with organs recovered from diseased pigs showed that *S. suis* was frequently isolated in conjunction with *A. pleuropneumoniae*, *P. multocida*, *E. coli* and many other microorganisms, although it is not indicated exactly how many of these cases were from lungs versus other internal organs/tissues [120, 121]. It has been reported that *S. suis* was isolated with many other bacterial pathogens in more than 75% of lungs displaying fibrino-hemorrhagic pneumonia [122]. *S. suis* and *P. multocida* were isolated together in almost 50% of lungs from animals suffering from respiratory clinical signs in China [123]. In addition, there was a reported case of concurrent lung infection of *S. suis* and *P. multocida* in conjunction with *Pneumocystis carinii* [124]. Using more sensitive PCR techniques, statistical associations were found in samples from lung tissues between PCR-positive results for *P. multocida* or *A. pleuropneumoniae* and *S. suis* [32]. Interestingly, a case of pneumonia in a sow with mixed infections of *S. suis* and *B. bronchiseptica* was also described, although *S. suis* causing disease in adult animals is extremely rare [125]. Another study indicated that almost 30% of affected lungs yielded common isolation of these two pathogens [126]. The association of both *S. suis* and *B. bronchiseptica* was further studied in in vitro and in vivo models (see below). Interesting, there is only one report indicating a possible correlation between *S. suis* and *M. hyopneumoniae* in the lungs [127].

The indication of the involvement of *S. suis* in mixed infections as a cause of pulmonary disease may also be found in other animal species. There was a reported case of acute death of a racehorse with clinical signs of pneumonia during transport in Japan [128]. *Pasteurella caballi*, *S. suis* and *Streptococcus zooepidemicus* were isolated from the lungs, indicating that the cause of pneumonia and subsequent death was due to the bacterial mixed infection of the lungs [128]. However, although these bacteria were isolated in high loads from lung and tracheo-bronchial lymph nodes, it was not possible to determine if they were the primary cause or secondary invaders [128]. Furthermore, the transport of the horses was lengthy and during winter months—two factors that may have created major environmental stress that enhanced bacterial growth and susceptibility to infection [128].

The isolation of *S. suis* with other bacterial pathogens in tissues other than lungs has also been reported. *S. suis* was isolated with *M. hyorhinis* or *M. hyopneumoniae* from the pericardium [129]. Another study reported mixed infections of *G. parasuis* and *S. suis* in cases of polyserositis [130]. Both pathogens were also detected together in more than 30% of samples, although the tissue/organ of origin (lungs versus internal organs) was not reported [131]. Finally, a case of abortion with dual isolation of *S. suis* and *Arcobacter spp* was described [132]. Mixed infections caused by different serotypes of *S. suis* should also be considered, as there have been recorded cases of meningitis and septicemia in a seven-week-old piglet caused by *S. suis* serotype 3 cultured from the spleen and bursa and a *S. suis* serotype 7 recovered from the cerebrospinal fluid [133]. It should be noted that all cases discussed in this section represent sporadic mixed bacterial infections without any clear indication of the specific role of co-infections in the development of disease.

### In vitro and in vivo studies on the interaction between *S. suis* and other bacterial pathogens

One in vitro study addressed a co-infection between *S. suis* and *G. parasuis* using NTPr cells and PAMs (Table 3) [54]. *G. parasuis* is a causative agent of Glässer’s disease in pigs. Similar to *S. suis*, low virulent strains colonize the upper respiratory epithelial surface of healthy animals, while high virulent strains can cross the epithelial barrier and cause mainly systemic disease and (probably) pneumonia (as a secondary invader) [134]. The disease is characterized by polyserositis and polyarthritis [134]. *G. parasuis* and *S. suis* infections also share similar clinical signs that frequently represent a challenge in the clinical differential diagnosis. The in vitro model using NTPr cells showed that low and high virulent strains of *S. suis* and *G. parasuis* have a different pattern of adhesion to and invasion of epithelial cells as single infections [54]. However, co-infection studies show limited in vitro interaction between the two bacterial species, which likely use different host receptors. Although it has been found that the capsular polysaccharide of *S. suis* serotype 2 has anti-phagocytic properties against a heterologous bacterial species, pre-treatment of PAMs with *S. suis* did not have a clear effect on the phagocytosis of *G. parasuis*. When inflammatory mediators produced by both epithelial and PAM co-infected cells were studied, an additive (but not synergistic) effect was observed. Virulence factors of *G. parasuis* and *S. suis* are not fully known and other conditions in respiratory mucosa may play an important role in *G. parasuis* and *S. suis* co-infections [7, 135].

**Table 3:** **Summary of the experimental conditions of** **S. suis** **co-infection with bacterial pathogens using in vitro models**

Pathogen studied	Type of cells or growth medium	Co-infection bacterial strain(s)	*S. suis* serotype 2 strain	Co-infection dose	Goal of the study	Conclusions	Refs.
*Glaesserella parasuis*	Neonatal tracheal porcine cell line (NTPr) cells	*G. parasuis* serotype 6 (low virulence) and serotype 5 (high virulence)	ST1 (high virulence) and ST25 (intermediate virulence)	Adhesion/invasion assays: 5 MOI (both bacterial species) Gene expression: 10 MOI (both bacterial species)	Adhesion and invasion; expression of inflammatory genes (qRT-PCR)	No effect	[54]
	Pulmonary alveolar macrophages	*G. parasuis* serotype 6 (low virulence) and serotype 5 (high virulence)	ST1 (high virulence) and ST25 (intermediate virulence)	Adhesion/invasion assays: 5 MOI (both bacterial species) Gene expression: 10 MOI (both bacterial species) *Phagocytosis: 100 MOI (both bacterial species) Expression of surface markers: 500 MOI/ 50 MOI for *G. parasuis*/*S. suis*	Adhesion/invasion, phagocytosis, inflammatory gene expression, and expression of surface markers	No effect	[54]
*Actinobacillus pleuropneumoniae (App)*	Medium for bacterial culture (no cells)	*App* (no further details on the strain)	No details	*App* and *S. suis* mixed in 1:1 ratio	Biofilm formation, antibiotic resistance, and expression of genes for virulent factors	**↑** Biofilm formation (*App*), antibiotic resistance and genes coding for different (putative) virulence factors for both pathogens	[136]
*Bordetella bronchiseptica*	Porcine precision-cut lung slice	Field strain of *B. bronchiseptica*	ST1 (high virulence)	10^4^ and 10^4^ CFU/well for *B. bronchiseptica* and *S. suis*, respectively	Effect of a pre-infection with *B. bronchiseptica* on adhesion, colonization and toxicity of *S. suis*	**↑** Bacterial adherence, colonization and cell toxicity	[137]

^*^Cells were infected with *S. suis* first and then with G*. parasuis;* ST: sequence type.

A recent report suggested that co-infections between *A. pleuropneumoniae* and *S. suis* are frequently found in China, although no data were presented [136]. However, the authors hypothesized that there are synergistic interactions between both pathogens. They reported that, when *A. pleuropneumoniae* was cultured with *S. suis* in culture media, biofilm formation by *A. pleuropneumoniae* was significantly increased. Moreover, compared to cultures with a single bacterium species, the antibiotic resistance, as well as the expression of genes coding for different (putative) virulence factors of both pathogens, were enhanced in the co-culture model. The authors suggest that the interspecies interactions between *S. suis* and *A. pleuropneumoniae* may be cooperative under specific conditions and may play an important role in disease progression and persistent infection [136]. It should be noted, however, that both pathogens affect piglets of different ages, with *A. pleuropneumoniae* mainly causing disease in grower-finisher animals and *S. suis* affecting mostly nursery piglets [36]. Indeed, the implications of the results obtained in the *S. suis*-*A. pleuropneumoniae* co-infection study should be carefully interpreted, as they probably do not reflect the situation in the field.

A last in vitro study became available very recently [137], where the interaction of *B. bronchiseptica* and *S. suis* was analyzed using the PCLS system previously reported (81). Interestingly, the authors established conditions where the pre-infection of *B. bronchiseptica* induced reduction of ciliary activity in the absence of significant toxicity. Under these conditions, it was shown that *S. suis* significantly increase adhesion and colonization, although both pathogens seem to adhere and colonize different areas of tissue. In addition, the increased adhesion of *S. suis* resulted in higher cytotoxicity due to the presence of suilysin [137]. Although not confirmed, in vivo experiments suggest that a pre-infection with *B. bronchiseptica* may promote not only adhesion but also invasion into deeper tissues (see below).

In vivo studies are also very limited (Table 4)***. ***There is one report in which *S. suis* and *Aerococcus viridans* were isolated together from a pig with meningitis in China [138]. *A. viridans* has been isolated mostly from swine and humans [139, 140]. However, because *A. viridans* is a bacterium that is present in the environment, it may simply be a contaminant in a sample. To examine the potential of *S. suis* and *A. viridans* interaction, Pan et al. used an intraperitoneal mouse model of co-infection using both bacterial strains recovered from the above-mentioned case of meningitis [138]. Results showed that the co-infection significantly increased systemic infection and acute meningitis [138]. Titers of *S. suis* were significantly increased in the lungs of co-infected mice, while titers of *A. viridans* in lungs remained at a low level [138]. It should be noticed that colonization of the lungs was probably the result of septicemia (due to the injection route) and does not indicate a respiratory infection. Further research is required to see if these results could be reproduced in the swine model and examine the cellular and molecular mechanisms involved in this type of co-infection. However, it is important to keep in mind that his type of co-infection is almost never observed in the field and that the presence of *A. viridans* in the original clinical sample could have simply represented a contamination.

**Table 4:** **Summary of the experimental conditions of** **S. suis** **co-infection with bacterial pathogens using in vivo models**

*S. suis* coinfection pathogen	Age of animals infected with 1st bacterial *spp*.	Age of animals infected with 2nd bacterial *spp*.	1st bacteria route of infection	2nd bacteria route of infection	1st bacteria and dose	2nd bacteria and dose	Conclusions	Refs.
*Aerococcus viridans*	Five-week-old BABL/c mice	Five-week-old BABL/c mice	IP	IP	10^3^ – 10^8^ CFU *S. suis* « novel serotype» (no further details on the strain)	10^3^ – 10^8^ CFU *A. viridians*	**↑** Mortality	[137]
*Bordetella bronchiseptica*	Five-day old germ-free piglets	Seven-day-old germ-free piglets	IN	IN	0.5 × 10^7^ CFU *B. bronchiseptica*	0.7 × 10^6^ CFU; serotype 2, ST1, different phenotypes	**↑** Clinical signs	[141]

IP: intraperitoneal injection; IN: intranasal inoculation; CFU: colony-forming units; ST: sequence type.

The most important in vivo study has been carried out with *S. suis* and *B. bronchiseptica* [141]. Interestingly, the original goal of the use of *B. bronchiseptica* in this study was simply to predispose pigs to the intranasal infection with *S. suis*. Indeed, as mentioned above, it is difficult to reproduce *S. suis* disease with conventional pigs using this route of infection. In this study, a *B. bronchiseptica* strain isolated from the nose of a pig with regressive atrophic rhinitis was used. In other words, the study was oriented to a local infection (nose) affecting the mucosa rather than a lower respiratory infection (lungs). Indeed, in further studies, acetic acid was used to replace *B. bronchiseptica* infection [33]. In the co-infection study, *S. suis* strains that were phenotypically different with regard to virulence markers were used. Only *S. suis* strains that harbored the proposed virulence markers were able to induce disease. Interestingly, *S. suis* was detected in the lungs with bronchopneumonia only when *B. bronchiseptica* (at higher concentrations) was also present. Indeed, *B. bronchiseptica* was recovered from the lungs of 19 animals, whereas *S. suis* was present in 8 samples. None of the samples contained *S. suis* only. The authors concluded that *S. suis* may be considered a secondary agent of pneumonia [142]. In vitro results recently obtained and discussed above may explain mechanisms involved in the interaction of both bacterial species [137].

The abundance of bacterial pathogens found in epidemiological association with *S. suis*, especially in pneumonia cases, is not followed by the appropriate number of co-infection studies to understand the role and mechanisms involved in such interactions. In vivo studies of co-infection of *S. suis* with other important bacterial pathogens are not available, and it is therefore difficult to speculate on the significance of each pathogen in the inception of disease.

## Limitations of *S. suis* co-infection studies

Generally speaking, the limitations of co-infection studies are well addressed by Saade et al. [17]. More specifically with regard to *S. suis*, although mixed infections studies may be taken into consideration, it is important to interpret the data on a real synergic effect in the field carefully, and reports from lungs recovered at the abattoir should be avoided. Indeed, the fact that *S. suis* is a normal inhabitant of the upper respiratory tract should not be neglected. Regarding in vitro models of co-infection, several have limitations and room for improvement. *S. suis* infection models using different immortalized or primary cells are still useful to study the mechanisms of gene expression and cellular pathways induced by viruses and bacteria and may shed light on the molecular interactions that occur during co-infections. It should be noted that the choice of cell type and nutritional components of the media may have a major impact on pathogen interactions, growth and survival. For example, the expression of certain metabolic and virulent bacterial genes by *S. suis* may be different or reduced in nutritionally rich media compared to in vivo-like environments [143]. To reproduce cell interactions in a more complex environment, the development of ALI and PCLS systems constitutes a major improvement to study *S. suis* co-infections [81, 82]. Enhanced in vitro systems such as three-dimensional organotypic tissue models that more closely resemble the 3D architecture, cellular composition and matrix complexity of the organ may be further developed. The adaptability of these tissue-engineered models to multiple pathogens suggests significant potential for infectious disease studies [86].

In many cases, the innate immune system plays an important role in the outcome of the co-infection and, as such, many studies focus on the interaction with the immune cells. In comparison with mouse or human immunology, there is still a lower number of immunological tools designed to detect and analyze swine immune cells [144]. However, since the field of swine immunology is evolving rapidly, we can expect that more sophisticated immunological tools will be developed in the near future and significantly expand our knowledge of the immune response induced during these co-infections.

In general, for many infectious agents, in vivo models of co-infections are sometimes difficult to reproduce [17]. This is even truer when referring to *S. suis*:

- Firstly, *S. suis* is a normal inhabitant and almost all conventional animals used in experimental infections are already colonized. Since there are no sensitive and specific validated tests (neither PCR nor serological tests) to evaluate whether animals are carriers of virulent strains, it is difficult to predict how the animals will behave after experimental infection. We have already recovered *S. suis* serotypes 1 and 14 isolates from the internal organs (one case for each serotype) of animals experimentally infected with a virulent serotype 2 strain, possibly due to a previous infection present in the animals’ herds of origin (unpublished observations). On the other hand, the use of caesarian-derived, colostrum-deprived piglets (highly susceptible to *S. suis*) constitutes a very useful tool to generate knowledge but somehow remains relatively far from the reality of the field.
- Another issue is that the routes of *S. suis* inoculation (even with virulent serotype 2 strains) are rather artificial (intravenous, intraperitoneal, intramuscular) due to the difficulties to reproduce the disease in conventional animals [16]. A seeder-to-naïve model, as developed for other infections such as *A. pleuropneumoniae* [145], has yet to be established. In co-infection studies, these procedures complicate the interpretation of the results, since the first pathogen frequently infects animals through the respiratory tract, and both pathogens should be evaluated under similar conditions.
- As mentioned, the virulence of serotype 2 strains from different geographical origins may be different [2], and almost no co-infection studies were conducted with well-characterized North American serotype 2 strains with lower virulence capacity when compared to their Eurasian counterparts.
- There are almost no studies with serotypes other than serotype 2, such as serotype 1/2 which is highly prevalent in North America. This is mainly due to the fact that experimental infection models with non-serotype 2 strains are rarely developed.
- Environmental and management factors may highly influence the development of the disease by *S. suis*. These factors are usually not present under experimental conditions in well-controlled research environments. In this regard, recent experiments on *S. suis* mucosal infection models tried to mimic, at least in part, the weaning stress that piglets experience under field conditions by applying several stressors, like transport and social stresses [4].

## Conclusion and future perspectives

Co-infections of *S. suis* with important swine pathogens have been addressed in recent studies, and new mechanisms of pathogen interactions of viral and bacterial pathogens have been proposed. However, owing to the importance given to co-infections in *S. suis*-associated diseases, the number of studies (mostly addressing mechanisms) is considerably low. In general, viral pathogens such as SIV may destroy the epithelial barrier and increase the permeability of the mucosal surface, while PRSSV may have a negative impact on the functions of pulmonary alveolar and intravascular macrophages and PCV-2 has a strong tropism for lymphoblasts. However, how these mechanisms actually affect *S. suis* pathogenicity is still debatable. Similarly, an increased inflammation (a hallmark of *S. suis* infections) caused by co-infection with other pathogens may also play an important role. However, most co-infection studies showing upregulations of inflammatory mediators were conducted in vitro and the data must still be validated in vivo within the natural host. Finally, data on co-infections (and mechanisms involved) of *S. suis* with other bacterial pathogens are mostly unavailable.

Saade et al. made general recommendations for future co-infection studies [17]. These recommendations may be adapted to *S. suis* co-infection studies: (i) authors should clearly indicate and characterize the *S. suis* strains used (e.g., serotype, sequence type, presence of virulence markers and other phenotypic characteristics); (ii) well-characterized *S. suis* strains of serotypes other than type 2 should also be used, especially in North American studies in which other serotypes (e.g., serotype 1/2) may be more prevalent than serotype 2; (iii) in in vivo studies, the route of infection should follow the natural infection of both pathogens, which should be able to interact in the same environment; (iv) since *S. suis* mainly causes disease in nursery piglets, co-infection studies should mostly address pathogens affecting animals at a similar age; (v) no test is specific or sensitive enough to monitor the status of *S. suis* infection in a herd. Authors should avoid referring to the use of *S. suis serotype 2-free animals* based on serological (no scientific test available) and/or tonsillar PCR evaluations, being the latter low sensitive. Although some genes involved in the susceptibility or resistance to *S. suis* disease have been suggested in mice [143], no study has addressed the issue in pigs, and it is therefore not possible to identify animals susceptible to the infection yet; (vi) authors should try to use animals from farms with no or limited clinical cases (and serotype different from the one to be evaluated) of *S. suis* where no prophylactic/metaphylactic procedures are in place at the nursery; (vii) re-isolation of *S. suis*, serotyping and, if possible, pathotyping are mandatory in experimental co-infection studies using conventional animals, since *S. suis* other than the strain used during the co-infection may be isolated from diseased animals, especially the lungs. Improved and more standardized models will generate new data on the intricate process of *S. suis* interactions with major swine pathogens.

Finally, there may be additional effects of a previous infection with a pathogen on a secondary *S. suis* infection. For example, it has been reported that a previous infection with PRRSV may alter the efficacy of ceftiofur treatment of *S. suis* co-infection [146]. Whether a previous virus infection affects the antibody and protective response of *S. suis* autogenous vaccines is also unknown [26].

## Abbreviations

ALI: Air–liquid culture system
BMDC: Bone marrow-derived dendritic cells
DCs: Dendritic cells
MDCK: Madin-Darby canine kidney cells
NTPr: Neonatal tracheal epithelial cells
PAMs: Pulmonary alveolar macrophages
PCLS: Precision-cut lung slice
PCV-2: Porcine circovirus 2
PCVAD: Porcine circovirus associated diseases
PCVD: Porcine circovirus diseases
PMWS: Postweaning multisystemic wasting syndrome
PRDC: Porcine respiratory disease complex
PRRSV: Porcine reproductive and respiratory syndrome virus
PRV: Pseudorabies virus
ST: Sequence type
SIV: Swine influenza virus
